# Assessing the feasibility and acceptability of Changing Health for the management of prediabetes: protocol for a pilot study of a digital behavioural intervention

**DOI:** 10.1186/s40814-019-0519-1

**Published:** 2019-11-26

**Authors:** Sophie Cassidy, Nduka Okwose, Jadine Scragg, David Houghton, Kirsten Ashley, Michael I. Trenell, Djordje G. Jakovljevic, Kate Hallsworth, Leah Avery

**Affiliations:** 1 Institute of Cellular Medicine, Newcastle University, Newcastle Upon Tyne, UK; 2 NIHR Innovation Observatory, Newcastle University, Newcastle Upon Tyne, UK; 3 The Liver Unit, Newcastle Upon Tyne Hospitals NHS Foundation Trust, Newcastle Upon Tyne, UK; 4 Centre for Rehabilitation, Exercise and Sports Sciences, School of Health & Social Care, Teesside University, Tees Valley, UK

**Keywords:** Prediabetes, Lifestyle behaviour change, Digital intervention, Diet, Physical activity, Weight loss

## Abstract

**Background:**

The prevalence of prediabetes is rapidly rising in the UK, largely associated with an increase in obesity. Lifestyle programmes that provide support to make and sustain dietary and physical activity behavioural changes are necessary to initiate and maintain weight loss. However, these programmes are often intensive and time consuming. Given the magnitude of the problem, there is a need for behavioural interventions that can be delivered at scale. Digital interventions can address some of the aforementioned issues. The primary aim of the present study is to assess the feasibility and acceptability of a digital intervention called Changing Health that provides structured education and lifestyle behaviour change support to adults with prediabetes.

**Methods:**

A single-group pilot study will be undertaken. We aim to recruit 40 participants with prediabetes defined by HbA1c or fasting plasma glucose (FPG), aged between 18 and 75 years with a BMI ≥ 25. Participants will receive the digital intervention (a mobile phone app incorporating structured education and behavioural tools to support lifestyle behaviour change) with the aim of losing and maintaining 5–6% of their baseline body weight. Each participant will receive 100 min of lifestyle coaching over the 9-month intervention period and will have continued access to the digital intervention. Clinical outcome measures will be collected during four visits to our clinical research facility: two visits at baseline, one visit at month 3, and one visit at month 9. These secondary outcome measures will include diet, physical activity, sleep, metabolic control, body composition, cardiorespiratory fitness, and cardiovascular function. To measure primary outcomes, an embedded qualitative study will be conducted to obtain data on feasibility and acceptability of the intervention.

**Discussion:**

This pilot study will establish whether Changing Health is feasible and acceptable to adults with prediabetes. Clinical outcome measures will provide estimates of variability to inform sample size calculations, and qualitative data generated will inform any necessary refinements to the intervention. This will provide a platform for a larger evaluation to assess the effectiveness of Changing Health for changing diet and physical activity to initiate and maintain weight loss in adults with prediabetes.

**Trial registration:**

ISRCTN Registry: ISRCTN69270299.

## Background

The prevalence of prediabetes in the UK is rapidly rising [1], and recent figures suggest that 12.3 million UK adults are currently at increased risk of type 2 diabetes [2]. Weight gain is one of the main risk factors for prediabetes, with obese individuals having a sevenfold increased risk of developing type 2 diabetes compared to healthy weight individuals [3]. Fortunately, these risk factors are modifiable. There is a growing body of evidence to demonstrate that effectively targeting diet and physical activity reduces diabetes incidence [4], with weight loss being the dominant predictor of diabetes risk reduction [5].

The National Health Service (NHS) diabetes prevention programme was officially launched in 2015 to support those at high risk of type 2 diabetes to make healthy lifestyle changes [6]. It consists of 13 education and exercise sessions, lasting between 1 and 2 h, most of which (at least 16 h) is delivered face-to-face by health professionals (e.g. healthcare assistants and nurses). Despite early evidence of weight loss [7] and patient uptake [8] which has exceeded expectations, there are a number of issues with this service. It is intensive and time consuming to deliver and requires a substantial time commitment from patients to attend regular face-to-face sessions which may hinder scalability for population use [9]. Digital interventions can offer a solution to these issues by providing structured education and lifestyle behaviour change support at scale, low cost [10] and with the opportunity to integrate wearables and other apps to maximise adherence and effectiveness [11]. The use of digital interventions may also help to address the issues of long waiting lists, and suboptimal engagement and attendance rates on programmes currently delivered face-to-face.

Although findings are mixed in terms of whether theory-based interventions are more effective than interventions that are not theory-based or informed, supporting lifestyle behaviour change with theory-informed behaviour change interventions has shown to be important for optimising long-term glycaemic control [12, 13] and weight loss [14]. However, the availability, uptake, and faithful delivery and receipt of such interventions in primary care are lacking [15]. To address this issue, we developed a multifaceted behaviour change intervention programme to increase physical activity in adults with type 2 diabetes called Movement as Medicine [16]. Primary care professionals (i.e. healthcare assistants, practice nurses, and GPs) were trained to deliver a behavioural intervention targeting increased physical activity during routine consultations and to provide long-term support using specific behaviour change techniques delivered during face-to-face consultations. Briefly, this intervention consisted of an online training programme for primary healthcare professionals that provided information content and skills-based training to successfully deliver the patient intervention. The patient intervention included a DVD providing narratives of the types of activity others had chosen to successfully manage their diabetes (behaviour change technique (BCT): providing information on the consequences of behaviour for the individual), paper-based resources to help facilitate a discussion about increasing activity levels (BCT: provide feedback on performance), and planning and monitoring resources including a pedometer (BCTs: action planning, self-monitoring). Full details of the intervention have been published previously [17]. This intervention was shown to be acceptable by healthcare professionals and patients, and feasible for delivery during routine primary care [18]; however, qualitative data highlighted the need for an intervention that targeted both physical activity and diet to initiate weight loss. It was also emphasised by healthcare professionals that an intervention targeting patients at risk of diabetes was required with an emphasis on weight loss. Given the time constraints in primary care, concerns were also raised about the time required to deliver the intervention to all of those who require support. Furthermore, a common barrier reported by patients was the requirement to attend face-to-face structured education sessions that are often scheduled at inconvenient times and clash with work and family commitments. This highlighted a need for an alternative, scalable solution. Data generated from the Movement as Medicine for Type 2 Diabetes trial was therefore used to inform the development of the Changing Health intervention to address the needs identified. Specifically, the scope of the intervention was expanded to target both diet and physical activity behaviours with an emphasis on weight loss. In addition, face-to-face delivery by primary healthcare professionals was replaced with a digital intervention with lifestyle behaviour coach support to address the concerns of healthcare professionals about the time taken to provide support to all those who need it. In terms of behavioural content, the specific behaviour change techniques incorporated in Movement as Medicine were represented digitally within the Changing Health app and lifestyle behaviour coaches were trained to use these techniques during telephone calls. The aim of this pilot study is to assess feasibility and acceptability of the Changing Health intervention with adults at risk of type 2 diabetes. More specifically, the primary objectives are as follows:

1. To assess the feasibility of recruitment including length of time required to complete participant recruitment and retention rates (i.e. is it feasible to recruit the number of participants required within the specified recruitment time period)
2. To assess acceptability of the intervention to people with prediabetes
3. To assess adherence to and completion of the intervention (i.e. do participants work through all aspects of the app and take part in all coaching sessions delivered by telephone)
4. To assess whether the intervention was delivered and received as intended (i.e. to assess fidelity of delivery of the intervention by recording lifestyle behaviour coaching calls)
5. To conduct a qualitative process evaluation with participants to identify barriers and enabling factors to completion of the programme

The secondary objectives are to estimate the variability in weight, physical activity, diet, sleep, metabolic control, body composition, gut health, cardiorespiratory fitness, and cardiovascular function, by generating interval estimates of the mean difference over time for each outcome measure. This will enable the statistical power calculations for a subsequent larger scale evaluation to assess the effectiveness of the intervention as part of routine clinical care.

## Methods

### Study design, setting, and ethics

This is a single-group pilot study. All four study visits will be undertaken in the Clinical Research Facility, Royal Victoria Infirmary, The Newcastle upon Tyne Hospitals NHS Foundation Trust, UK. The intervention includes a mobile phone application which participants can access in their own time and setting. Ethical approval (protocol version 7.0) was granted by Preston Research Ethics Committee (17/NW/0130). NHS Research and Development approval was granted by the Newcastle upon Tyne Hospitals NHS Foundation Trust. The study was subsequently adopted and will receive support from the Primary Care Research Network (PCRN).

### Sampling and recruitment

A sample size of *N* = 40 was agreed in accordance with published guidance for pilot studies and allows for drop-outs [19]. Local primary care practices will be recruited through the North East and North Cumbria Clinical Research Network who help identify and approach participant identification centres (PICs). Those interested will be recruited on a first come first serve basis. Recruitment of participants will be facilitated by recruited primary care practices who will mail out invitations to patients meeting the study eligibility criteria (Table 1). These patients will be asked to contact a member of the study team if they are interested in taking part. During an initial screening visit, written informed consent will be obtained by a member of the study team. This will then be followed by a complete medical history, full body examination, and other clinical measures (see Table 2) which will determine if the patient is eligible to take part.

**Table 1:** Patient eligibility criteria

Inclusion criteria	
• Prediabetes based on primary care record of HbA1c 5.7–6.4% (39–47 mmol/mol) and/or FPG 5.6–6.9 mmol/L, in the past 12 months (see above for discrepancies between blood results)	
• No previous diagnosis of type 2 diabetes	
• Age ≥ 18–75 years	
• BMI ≥ 25 kg/m^2^	
• Weight stable for the past 6 months	
• Access to and able to use a smart mobile phone	
• Willing and able to provide written informed consent	
• Willing to undertake study activities	
Exclusion criteria	
• Inability to speak or read English without the assistance of an interpreter	
• Contraindications to exercise determined during exercise screening	
• Contraindications to weight loss	
• Mental or physical incapacity which makes self-management inappropriate	
• Pregnancy, planning pregnancy, or lactating	
• Unable to meaningfully participate for the full duration of the study	
• Participated in an intervention research study within the last 6 months

**Table 2:** Schedule of enrolment and assessments

Study phase	Baseline 1 (screening) (month 0)	Baseline 2 (month 0)	Mid visit (month 3)	End visit (month 9)
Week	− 1	0	13	37
Visit number	1	2	3	4
Inclusion/exclusion criteria	X	X	X	X
Informed consent	X			
Medical history	X			
Physical examination	X		X	X
Exercise stress test/cardiac and vascular function		X	X	X
Intake 24	X		X	X
Physical activity	X		X	X
Stool/urine sample	X		X	X
Fasting blood sample	X		X	X
Oral glucose tolerance test	X		X	X
Skin swab	X		X	X
Body composition/waist:hip	X		X	X

### Inclusion/exclusion criteria

The inclusion and exclusion criteria can be seen in Table 1. An HbA1c of 5.7–6.4% (39–47 mmol/mol) and/or fasting plasma glucose (FPG) of 5.6–6.9 mmol/L from primary care records will be used for diagnosis of prediabetes and type 2 diabetes. HbA1c does not necessitate fasting; therefore, it is the most practical measure to use when recruiting from GP practices. A HbA1c in the required range taken in the past 12 months will be used, or FPG if this is the only measure available from medical records. As part of the clinical testing (four visits to the lab), a number of blood tests will be performed. We therefore expect discrepancies between our blood results and those recorded in primary care, due to different testing methods and inherent biological variability. An update from the NHS DPP has highlighted a number of issues which may arise [20], and these will be resolved as described below:

1. *Discrepancy between referral test result and screening results.* The result from referral should be used to determine entry into the study. If the screening visit result suggests an individual has moved into the type 2 diabetes range, they will be referred back to their GP. If the patients’ HbA1c is ≥ 52 mmol/mol, they will not be considered eligible. Those with a value between 48 and 51 mmol/mol can elect to remain in the study, due to the variability around the HbA1c measurement.
2. *Inconsistent results from HbA1*_*c*_ *and FPG on referral.* If either reading is in the type 2 diabetes range, patients will not be eligible for inclusion.

### Intervention

Each participant will receive an email with instructions on how to access the app. Once this email is sent, the time taken for the participant to login will be monitored. In the instance where the app has not been accessed within a week of the email being sent, a lifestyle behaviour coach will contact the participant to ask if they require support to login and use the app. Participants will have continuous access to the Changing Health intervention for a period of 9 months. The intervention includes a mobile phone app with lifestyle behaviour coach support delivered by telephone. Participants will be supported to lose 5–6% of their baseline body weight over a 9-month time period. In a situation where participants achieve this target earlier, they will be supported to either (i) maintain weight loss or (ii) continue to lose weight should they wish. The mobile phone app provides structured education on a number of topics (see Table 3) delivered using a range of formats including animations, illustrated articles, and interactive features. Participants will be required to complete the structured education content before they are given access to a lifestyle behaviour coach. Coaching appointments can be pre-booked using a live calendar function linked to each coach’s availability. In addition to the structured education content, the app contains a range of behavioural features that enable participants to set goals, plan, and monitor their progress. For example, diet can be monitored using a photo diary via the camera of the smartphone, and daily physical activity levels can be monitored using the smartphone’s built-in step counting function. Lifestyle behaviour coaches will support participants to set realistic and meaningful dietary and physical activity goals, and they will be encouraged to log their weight on a weekly basis. The intervention is underpinned by the Health Action Process Approach [21] and incorporates a range of behaviour change techniques (e.g. information about health consequences, goal setting behaviour, goal setting outcomes, self-monitoring outcomes of behaviour) in accordance with the behaviour change technique (BCT) taxonomy V1 [21] to facilitate operationalisation of the behavioural constructs of the model (see Table 3 for a summary of behaviour change techniques of the intervention). The lifestyle behaviour coach will have access to all data recorded by each participant (e.g. goals, plans, and self-monitoring data) and will use this to inform the structure and delivery of each telephone coaching appointment. The coach will be able to view progress and will offer up to 1 × 20 min (first telephone call) and subsequent calls of 8 × 10 min over the duration of the programme. Participants will be able to choose the frequency of lifestyle coaching appointments up to a maximum of 100 min. Lifestyle behaviour coaches are individuals with qualifications in health psychology, sports and exercise science, nutrition, or public health and/or who have experience of working with people to support lifestyle behaviour change. They were recruited by Changing Health Ltd. on this basis, and each has received training delivered by a Chartered Health Psychologist with expertise in health behaviour change. Training was delivered face-to-face in a group setting over four consecutive days and covered information topics such as ‘what is prediabetes’ and ‘the use of diet and physical activity to prevent type 2 diabetes’, and focussed on the use of brief motivational techniques and delivery of behaviour change techniques in accordance with the content and underpinning theory of the intervention. A series of role play activities were undertaken to practice delivery of the intervention, and feedback was provided on performance.

**Table 3:** Modules within the education content of the app

Module	Purpose	Videos (V) + articles (A)	Behaviour change techniques incorporated
Understanding diabetes	• Explaining how type 2 diabetes affects the body • Making clear which risk factors are modifiable • Dispelling diabetes myths • Making clear that change is possible	V1—What is type 2 diabetes A1—What is prediabetes A2—Risk factors for diabetes A3—The role of your genes in diabetes risk V2—Managing your risk of type 2 diabetes A4—Understanding your test results A5—Why do not all bigger people get type 2 diabetes	• Information about health consequences
Steps to success	• Introducing the most important behaviour change techniques for risk modification • Providing a framework for successful lifestyle change • Introducing the concept of lifestyle coaching • Providing motivation to continue with the learning programme in order to gain access to a lifestyle coach	V1—Maximising your chance of success A1—Five essential tips to goal setting A2—Using ‘self-talk’ A3—Getting started with coaching	• Goal setting (behaviour) • Goal setting (outcome) • Self-monitoring of outcomes of behaviour • Action planning • Social support (unspecified) • Barrier identification • Time management • Self-talk • Feedback on behaviour • Review behaviour goals • Follow-up prompts
Understanding food	• Breaking down the food we eat into its component parts • Explaining how each of those parts affects glucose control	A1—Eating to feel full A2—Understanding fat in your diet V1—How eating affects your weight V2—Carbs in your diet A3—How big is ‘a portion’? A4—Top ten food myths	• Information about health consequences
Changing your diet	• Overview of three eating approaches that have been proven to be effective for improving glucose control • Interactive tools to support dietary change	V1—Finding the right diet for you V2—Low carb approach V3—Intermittent fasting V4—Mediterranean diet V5—Supermarket tour A1—Using prompts and cues A2—Recipe ideas for the low carb diet approach A3—More about the low carb diet approach A4—More about the Mediterranean diet A5—More about intermittent fasting	• Information about health consequences • Prompts/cues
Getting active	• The role of physical activity in weight loss and glucose control • Testing knowledge of activity recommendations • Introducing types of activity most effective for weight loss and improved glucose control	V1—How our changing lifestyles contribute to weight gain V2—Combining exercise and diet A1—Why combining works best A2—Aerobic and resistance fitness A3—Could HIIT be the right fit? A4—The benefits of resistance exercise Plugin: Set an activity goal	• Self-monitoring of behaviour • Information about health consequences • Provide instruction

### Primary outcomes

A mixed methods approach will be adopted to establish feasibility and acceptability. As such, we will collect data on feasibility and acceptability as outlined below.

#### Feasibility

Feasibility will be assessed by collecting data on the following:

- Recruitment and retention rates
- Adherence rates
- Time required to recruit to target
- Number of eligible participants required to recruit required sample size
- Rate of completion of the intervention (i.e. number of participants who access and complete all aspects of the intervention including lifestyle coach support)
- Feasibility of testing procedures and data collection methods, including completion rates

#### Fidelity assessment

All lifestyle behaviour coach telephone calls will be audio recorded throughout the intervention period to facilitate fidelity of delivery assessment (i.e. to determine whether it is feasible to deliver the intervention as intended). Recordings will be independently coded by two members of the research team using a checklist of intervention content. Inter-rater reliability will be assessed between coders using Krippendorff’s alpha [22]. We will also collect data on the number of times the mobile phone app is accessed by each participant and areas of the app most frequently accessed.

#### Acceptability

Acceptability of the intervention and study procedures will be assessed both quantitatively and qualitatively. Quantitative data will be collected to investigate (i) the number of times participants login to the app to access the education content and behavioural tools, and over what time period (i.e. whether access is within a discrete period of time or over the entire intervention period), and (ii) whether participants make an initial and subsequent lifestyle behaviour coach appointments over the duration of the intervention. An embedded qualitative study will obtain patients’ views and experiences of the intervention, including what they perceive to be barriers and facilitators to using it. In accordance with published guidance and similar research we have conducted [18, 23], and to allow for data saturation, at least 10 and up to 16 participants will be interviewed [24]. Topic guides will be used to conduct interviews (see Additional file 1 for an example topic guide). We will also record the uptake of lifestyle coaching to make an assessment of acceptability of the service.

### Secondary outcomes

Although this study is not powered to detect clinically significant changes in weight and clinical outcome measures, we will collect these data on each outcome respectively to generate interval estimates of the change and to also determine whether the testing components that would be used in a larger evaluation are feasible.

Participants will be asked to attend the Clinical Research Facility on four separate visits: two visits at baseline, one visit at month 3, and one visit at month 9. There are two baseline visits to prevent participants attending one prolonged visit. A number of physiological variables will be measured across the four visits (see Table 2), in the following categories:

1. *Metabolic control, weight, and body composition.* A 75-g oral glucose tolerance test will be performed, and plasma glucose and insulin will be measured over a 2-h period, so that insulin resistance and beta cell function, and area under the glucose curve can be predicted. Fasting plasma samples will be analysed for alanine phosphatase, alanine transaminase, aspartate aminotransferase, total cholesterol, triacylglycerols, and HbA1c. Weight and body composition will be measured using air displacement plethysmography (BodPod, Life Measurement, CA, USA).
2. *Cardiorespiratory fitness.* A medical history, physical examination, and 12-lead electrocardiogram will be performed prior to a maximal cardiopulmonary exercise test. The test will include a ramped exercise protocol using an electronically braked semi-recumbent cycle ergometer (Corival Lode, Groningen, The Netherlands) and gas exchange to measure peak oxygen consumption (VO_2peak_).
3. *Cardiovascular function.* Cardiac and vascular parameters will be assessed at rest (for 20 min) and during cardiopulmonary exercise testing using non-invasive methods for cardiac output, heart rate and blood pressure variability (TaskForce and Smart Cardia), and arterial stiffness (SphygmoCor).
4. *Physical activity, sleep + diet.* Physical activity and sleep will be measured using a 7-day accelerometer worn on the wrist (GeneActiv). Dietary patterns will be collected using Intake24 (https://intake24.co.uk/) which is a validated dietary recall method of dietary assessment, which requires individuals to input their food intake with accurate image-based portion size estimation over 3 days.
5. *Other.* Participants will be asked to provide a stool and urine sample for microbiome and metabolomic analysis for the assessment of bacterial flora and metabolites present in urine. A simple skin swab that assesses skin damage and tests for mitochondrial (mDNA) dysfunction will be performed.

### Criteria to proceed to a larger evaluation

A decision to proceed to a larger scale evaluation will be based on recruitment, retention, outcome data generated (amount of data obtained and the outcome of the data obtained), intervention acceptability and feasibility, and fidelity of delivery [25]. If we fail to recruit 80% of the target sample size, screening logs will be assessed to determine whether insufficient participants were approached/screened, whether insufficient participants met eligibility criteria, and from those eligible, whether they subsequently agreed to participate. Based on these findings, we will establish whether to proceed with a larger evaluation if the intervention was found to be both feasible to deliver and acceptable to participants following minor modification if required. Outcome data for at least 80% of participants at the 9-month time point will be required to help inform our decision on whether we should proceed to a larger trial (i.e. whether it is feasible to collect the data required and findings from the data once analysed).

### Analysis

#### Quantitative data analysis

Data from this pilot study will be descriptive with outcomes being interval estimates of variables relating to feasibility and acceptability. As this is a pilot study, the level of missing data will be documented but no imputation will be undertaken.

#### Qualitative data analysis

All interviews will be audio recorded and transcribed verbatim. Transcripts will be analysed using the Theoretical Domains Framework (TDF) [26]. The TDF was developed to simplify and integrate 33 behaviour change theories and 128 key theoretical constructs related to behaviour change. These were synthesised into a single framework to allow an assessment of behaviours. The TDF originally comprised of 12 domains, which was subsequently validated and refined to 14 domains. These are knowledge; skills; social/professional role and identity; beliefs about capabilities; optimism; beliefs about consequences; reinforcement; intentions; goals; memory, attention, and decision processes; environmental context and resources; social influences; emotion; and behavioural regulation. The 14 domain framework will be used for the purpose of this study.

A two-stage process will be followed in order to analyse interview transcripts. The first interview transcript will be pilot-coded independently by two researchers to agree a coding strategy (i.e. to ensure both researchers are coding consistently and to discuss and resolve any difficulties when applying the TDF). Initial findings of the pilot transcript will be discussed before coding the remaining transcripts. Secondly, data from the remaining transcripts will be independently coded by the same two researchers. This will involve reading and re-reading transcripts, coding the content into themes and subthemes, and mapping these, with supporting direct quotes, to an appropriate theoretical domain of the TDF.

## Discussion

Providing patients with structured education and lifestyle behaviour change support is essential to help prevent the onset of type 2 diabetes in those at risk [4]. Digital technology provides a means of delivering education and long-term lifestyle behaviour change support at scale and at a lower unit cost. Recent evidence suggests that behaviour change interventions provided through brief remote support can be effective for weight loss [27]. This study aims to assess feasibility and acceptability of the behavioural intervention Changing Health that targets diet and physical activity behaviours to initiate weight loss and support long-term weight loss maintenance in adults with prediabetes.

Targeting lifestyle behaviour has shown to lead to weight loss and a reduction in diabetes incidence [4]. The Changing Health intervention is underpinned by theory (Health Action Process Approach) and was systematically developed, informed by research evidence [12, 13, 16] to operationalise the constructs of the theory using specific behaviour change techniques. These techniques target motivation to support intention formation and volition to support enactment and maintenance of behaviour change. Lifestyle behaviour coaches are an important intervention feature/component to support meaningful and realistic goal setting, action planning, and coping planning and to facilitate adherence to the digital intervention. A mixed methods design will enable us to identify (i) whether lifestyle coaches can deliver intervention components as intended, and if so whether faithful delivery of these components leads to a change in lifestyle behaviours (diet and physical activity behaviour), and (ii) to determine whether the intervention components are acceptable to individuals with prediabetes. We will also assess whether recruitment to target is feasible and whether patients are willing to complete the intervention.

Weight loss is key to prevent type 2 diabetes; however, estimates suggest that only 20% of overweight individuals are successful at long-term weight loss [9]. Although the study is not powered to detect changes in weight and other clinical outcomes, we will measure these to provide calculations to inform a larger evaluation. We will explore the potential impact of the intervention on cardiorespiratory fitness, which is strongly linked to mortality and has also been shown to predict individual response to lifestyle intervention. The impact on metabolic control, cardiovascular function, and gut health will also be explored as weight loss leads to improvements in such variables [28].

The results from this pilot study will help to explore whether this digital intervention (Changing Health) involving structured education and lifestyle behaviour coach support is feasible and acceptable for adults with prediabetes. This will lay the foundations for a larger evaluation to determine whether this intervention is effective at scale for long-term weight loss and preventing type 2 diabetes in those at risk.

### Trial status

Recruitment will begin on July 2018 and be completed on December 2019. Protocol v7.0, 15 March 2018.

## Supplementary information

**Additional file 1.** Interview questions.

## Data Availability

The datasets generated and/or analysed during the current study are available from the corresponding author on reasonable request.

## Abbreviations

FPG: Fasting plasma glucose
OGTT: Oral glucose tolerance test
VO_2peak_: Peak oxygen consumption
