# Supplementary material for: Assessing the feasibility and acceptability of Changing Health for the management of prediabetes: protocol for a pilot study of a digital behavioural intervention
Source: Pilot Feasibility Stud. 2019 Nov 26;5:139. doi: 10.1186/s40814-019-0519-1 (PMC6878649; doi:10.1186/s40814-019-0519-1)
Supplement: Supplementary file 1 — Additional file 1. Interview questions. [file 40814_2019_519_MOESM1_ESM.docx]

# Additional file 1

**Topic Guide**

**Interview questions**

1. Before joining the programme, what did you expect from it?
2. How does what you expected from the programme differ from what you have received?
3. What do you want to achieve from the programme? [weight loss; improvements in diabetes; something else?]
4. What has been your experience of the dietary approaches suggested? [Prompts: have you attempted more than one approach? Which approach have you found to be most/least useful? How has the dietary approach affected your mood, motivation? Have you tried any of these in the past?]
5. Do you have any tips that you could give to others for sticking to the dietary approach you have chosen?
6. How successful do you believe the programme has been for you so far? [Prompts: weight lost, weight loss maintained; use of tools within the programme; support provided from the programme]
7. How satisfied are you with the programme so far? [Prompts: weight lost, ease of following, dietary approaches, type of support; energy levels]
8. Have you noticed any changes in the way you think about weight loss and keeping the weight off as the weeks go by?
9. What has been the most useful part of the programme?
10. What has been the least useful part of the programme?
11. Have you experienced any lapses while in the programme?

- [If yes] Can you tell me more about specific situation/s when this happened? What did it feel like after you lapsed? Has anything changed since the lapse? (for example did you put any measures in place to reduce the chance of it happening again?)
- [If not] What helped you successfully continue with the regime/ overcome your temptations? Did you have a strategy from the start? If so would you mind describing it? How did you feel like after you resisted possible temptations? Has anything changed since then?

1. Has anything in your life changed since you started the programme? [Prompts: change in circumstances that has positively or negatively affected your progress; physiological or psychological changes?]
2. How useful has the programme been for changing your lifetsyle long-term?
